# Translation of Data from Animal Models of Cancer to Immunotherapy of Breast Cancer and Chronic Lymphocytic Leukemia

**DOI:** 10.3390/genes15030292

**Published:** 2024-02-25

**Authors:** Reginald Gorczynski

**Affiliations:** Institute of Medical Science, Department of Immunology and Surgery, University of Toronto, C/O 429 Drewry Avenue, Toronto, ON M2R 2K6, Canada; reg.gorczynski@utoronto.ca

**Keywords:** breast cancer, lymphoma, immunotherapy, cytokines, gene-modified mice

## Abstract

The field of clinical oncology has been revolutionized over the past decade with the introduction of many new immunotherapies the existence of which have depended to a large extent on experimentation with both in vitro analysis and the use of various animal models, including gene-modified mice. The discussion below will review my own laboratory’s studies, along with those of others in the field, on cancer immunotherapy. Our own studies have predominantly dwelt on two models of malignancy, namely a solid tumor model (breast cancer) and lymphoma. The data from our own laboratory, and that of other scientists, highlights the novel information so obtained, and the evidence that application of such information has already had an impact on immunotherapy of human oncologic diseases

## 1. Introduction

The expansion of the application of novel therapies in the field of cancer treatment over the past several decades has been built upon an improved understanding of the basic biology of host:cancer cell interactions using animal model systems, and more recently on the manipulation of those interactions using immunotherapy and chemotherapy, to improve cancer growth control and eradication. A recent review [1] has provided a detailed discussion of the regulation of tumor immunity by manipulating the level of expression and engagement of inhibitory molecules and their receptors in the immune system [2,3,4,5], as well as the activation of such inhibitory ligand:receptors, generally referred to as checkpoint blockade [6,7,8]. In particular, it is known that in animal models, and now in humans, reversal of the checkpoint blockade releases the activation of anti-tumor responses [9,10,11]. The targeting of a novel ligand:receptor dyad, CD200:CD200R, in regulation of tumor growth control has been a primary focus of the work in my laboratory, and again studies in animal models, discussed in depth below, have highlighted the therapeutic potential of such targeting in both solid tumors and B-cell malignancies [12].

Other technologies, again first tested in animal models, have also been adapted to improve anti-tumor immunity. As but one such example, chimeric antigen receptor (CAR)-T-cell therapy has expanded traditional cancer treatments that previously used nonspecific drugs and monoclonal antibodies to target tumor cells, and attempts to leverage specific host immune system’s T-cells to recognize and attack tumor cells. T-cells are isolated from individual patients, modified to target tumor-associated antigens, and with FDA approval have been used to treat B-cell acute lymphoblastic leukemia, large B-cell lymphoma, and multiple myeloma [13]. While successfully used for many blood cell cancers, CAR-T technology has had limited success in solid tumors, likely because of many confounding issues including a lack of defined tumor-associated antigens; hypoxia in the tumor mass; immunosuppression within the tumor micro environments; reactive oxygen species; and limited T-cell infiltration into the tumor mass. A primary goal of current research in CAR-T cell therapy is thus to identify reliable tumor-associated antigens and develop cost-effective, tumor microenvironment-specific CAR-T cells for solid tumors. 

As mentioned above, and discussed in more detail below, my own laboratory has combined the use of CD200:CD200R immunotherapy with cancer vaccines as an immunotherapeutic approach to blood cell malignancies and breast cancer in animal models, stimulating anti-tumor immunity with tumor antigens delivered in the form of whole cells [14,15]. It is important to appreciate that peptides, nucleic acids, etc., have been used in vaccines by others [16]. Such vaccines have also incorporated other, cytokine-based approaches, to overcome immune suppression associated with the tumor/tumor microenvironment (TME) [17,18]. This will become apparent in a discussion of our work (below) from in vivo/in vitro studies, using first human chronic lymphocytic leukemia (CLL) cells growing in NOD-SCIDγ_c_^null^ mice, and then, subsequently, mouse breast cancer cell models [19,20].

Patient-derived tumor xenograft (PDX) models [21], which involve the transplantation of tumor tissues derived from cancer patients into immunodeficient mice, have also become a prominent weapon in the armamentarium fostering translational research, particularly for solid tumors. Initially, orthotopic xenograft (PDOX) models were used, where tumor tissue was implanted in mice in anatomical locations that corresponded to that in the patient. More recently, given the limitations imposed by the advanced surgical techniques and resource-intensive imaging technologies needed for PDOX, humanized mouse models, as well as zebrafish models, have come into vogue. In humanized mouse models, immunodeficient animals are manipulated to contain a human immune environment, thus allowing the study of a more “natural” interplay between a human tumor graft and the host immune system. The zebrafish patient-derived tumor xenografts (zPDX) and ex vivo patient-derived organoid (PDO) models have been particularly used as cancer drug discovery technologies. Both represent novel personalized medical animal models, providing a cost-effective approach for rapid drug testing in systems which attempt to mimic the in vivo environment and preserve individual tumor-related information for patients. 

It has been recognized for many years that a primary limitation to the eradication of solid tumors represents the failure not of control of local growth, but of metastatic spread [22]. Both the evolution of resistance to therapy and the development of metastasis are multi-step processes whereby tumor cells, following cell-intrinsic genetic and/or epigenetic adaptations, overcome a number of environmental and selection hurdles to acquire immune evasion mechanisms, invasive and migratory capabilities, altered interactions with stromal cells, and maintenance of self-renewal capacity. We have successfully used murine models of breast cancer to explore some of the features which highlight the mechanisms involved in the control of local and metastatic tumor growth, as well as therapeutic resistance. Moreover, we have compared work in these in vivo models with data from an ex vivo 3D model of murine breast cancer growth to examine whether this can be used to model interactions of tumor cells within a tumor microenvironment [17,18]. It is acknowledged that while 2D in vitro models provide cost-effective and reproducible systems for study, cells in 2D co-cultures form a monolayer, and thus these models cannot accurately mimic the TME’s complex cellular interactions and signaling pathways, which take place in vivo in a 3D environment with different cellular morphology and polarization of the TME components. In a final section below, data from a novel 3D in vitro model we used in a breast cancer model [20] will be discussed and characterized for their utility in studying the complex interactions within the TME, while reproducing many of the phenomena already described from in vivo studies. 

## 2. Use of Xenotransplant Animal Models and In Vitro Studies in CLL (See Refs. [15,23] for Details)

### 2.1. Methodology … CLL Studies

*Mice:* NOD-SCIDγ_c_^null^ mice were bred and maintained under sterile conditions at the Toronto Medical Discovery Tower, MaRs Centre. All mice were used at 8–13 weeks of age, using a protocol approved by an institutional ethics review board. A minimum of 3 mice/group were used in all studies.

*Human splenocytes and CLL cells:* These were obtained from Sunnybrook Health Science Centre, under an ethics Review Board protocol from Dr D Spaner, gratefully acknowledged as a key collaborator in all of these studies. Cells were processed as described elsewhere [15,23] and frozen in liquid nitrogen for later use. For in vivo studies in NOD.SCID mice, aliquots of splenocytes were rapidly thawed at 37 °C, washed in PBS, and cell aggregates were separated by centrifugation on Ficoll-Paque PLUS gradients. Cells recovered after centrifugation were resuspended in PBS at appropriate concentrations for injection. In other studies, the peripheral blood of CLL patients was used for the reconstitution of mice, and CD19^+^CD5^+^ CLL cells were purified from fresh blood as previously described [23]. All protocols were approved by institutional review boards.

#### 2.1.1. Human Plasma and Engraftment of Human CLL in NOD-SCIDγ_c_^null^ Mice

Plasma from CLL patients was obtained at routine clinical follow-up and stored at −20 °C. For in vivo studies in NOD.SCID mice, plasma from a group of patients at late disease stage (Rai Stage III-IV), and/or with high white cell count, were pooled into batches (>8 donors/batch). Control plasma used was pooled from healthy volunteers. sCD200 levels in all plasma samples were assessed by CD200 ELISA [23]. sCD200 levels in pooled normal plasma were in the range 0.5 ± 0.2 ng/mL, while in various pooled CLL plasma batches levels were ~10-fold higher (5 ± 1.3 ng/mL). Rat anti-hCD200 monoclonal antibodies (1B9: now available from Cedarlane Labs. Hornby, ON, Canada) and Fab fragments have been described before [15,23]. 

On the day of experimentation, mice received 245 Rads of γ-irradiation, followed by 1 × 10^8^ human CLL cells ip, and 0.8 mL of pooled CLL plasma or control plasma, also given ip. Subsequent infusions of CLL plasma or control plasma were performed bi-weekly throughout the course of study. Animals were sacrificed at various time points to assess for CLL engraftment, harvesting spleen, bone marrow, and peritoneal cells from individual mice. 

#### 2.1.2. CD200 Blockade and T-Cell Depletion In Vivo Studies

For CD200 blockade experiments, mice were randomly assigned into 3 groups after infusion of human splenocytes. Two of the three groups received sCD200^hi^ CLL plasma while a third group received sCD200 absorbed CLL plasma. Of the groups that received CLL plasma, 1 group also received 3 doses of 50 μg (iv) of Fab anti-CD200 mAb at 72-h intervals. 

For the T-cell depletion experiments, mice received human splenocytes and sCD200^hi^ CLL plasma, or sCD200 absorbed CLL plasma. The following day, animals were randomly assigned to receive 20 μg OKT3 (anti-CD3; purchased from Ortho-McNeil Pharmaceuticals (Raritan, NJ, USA) antibody iv, with 2 additional infusions at 72-h intervals. 

#### 2.1.3. FACS Analyses

CD200 cell surface staining was performed using a rat anti-CD200 mAb. Multi-color FACS analyses were performed to characterize engrafted human cells. The optimal concentration of antibody for staining was individually determined for each antibody. Single-color controls were included in each experiment for compensation purposes, and all samples were analyzed in a Coulter FC500 flow cytometer. The one-step staining of cells from reconstituted mice was conducted according to the manufacturer’s instruction, using CD45-PE-Cy7, CD19-PE-Cy5, CD5APC, CD4-FITC, CD8-PE (Biolegend, San Diego, CA, USA).

*In vitro studies with CLL and BMMCs:* Primary human bone marrow mesenchymal stem cells were purchased from Lonza (Lonza, Basel, Switzerland) [15,19] and used in studies of growth of CLL. For studies using CLL cells as a tumor vaccine, CLL cells (1 × 10^6^/mL) were cultured in AIM-V medium and treated with PMA(30 ng/mL) (Sigma-Aldrich, St. Louis, MO, USA), IL-2(500 U/mL) (Biolegend, San Diego, CA, USA), and a TLR-7 agonist (Imiquimod) (3 µg/mL) (Sigma-Aldrich, St. Louis, MO, USA), with or without the addition of ionomycin (2 µmol/L). Cells were harvested after 5 days, washed, and resuspended in 0.5 mL regular PBS prior to injection into mice which were later challenged with live CLL cells (below).

#### 2.1.4. Cell Culture and Transwell Assay [19]

CLL cells were cultured in AIM-V medium (Life technologies, Carlsbad, CA, USA). Bone marrow mesenchymal stem cells (BMMSCs) were cultured in mesenchymal stem cell growth medium (MSCGM: Lonza, Basel, Switzerland). Both cell populations were cultured in 10-cm dishes or 24-well plates at 37 °C in a 5% CO_2_ incubator. To prepare BMMSC conditioned medium, BMMSCs were cultured in MSCGM to 70% confluence, with the medium then replaced with AIM-V medium for 24 h. The conditioned AIM-V medium was used in both in vitro/in vivo experiments. 

In co-cultures, 0.5 mL of CLL cell suspension (5 × 10^6^/mL) was incubated on a layer of adherent BMMSCs. For indirect co-culture, the same number of CLL cells was incubated in a transwell insert with 0.4-μm filters (Fisher scientific, Waltham, MA, USA) with mesenchymal cells cultured on the bottom of a 24-well plate. 10 μg/mL Actemra (Roche, Basel, Switzerland) or 0.5 μg/mL anti-IL8 antibodies (R&D System Inc., Minneapolis, MN, USA) were added to wells in some experiments. In other studies, 10 ng/mL hrIL-6 or 10 ng/mL hrIL-17A (Biolegend, San Diego, CA, USA) was used to stimulate CLL cells, BMMSCs, or the co-cultures of these two cells [19]. 

*Cytokine measurements in vitro/in serum*: A Multi-Analyte ELISArray kit (Qiagen, Venlo, The Netherlands) was used to check multiple cytokines in the culture supernatant of BMMSCs or CLL cells alone, or in co-culture. Levels of IL-1α, IL-1β, IL-2, IL-4, IL-6, IL-8, IL-10, IL-12, IL-17A, IFN-γ, TNF-α, and GM-CSF were measured. CLL plasma was collected from consenting CLL patients at routine clinic follow-up. Plasma from healthy donors was obtained from 10 sex and age matched individuals. sCD200, IL-6, and IL-17A levels in the CLL plasma and culture supernatant were tested with human ELISA kits (Biolegend, SD, USA). 

*Statistics:* Within experiments, comparison was made between groups using ANOVA, with subsequent paired Student’s *t*-tests as indicated. 

### 2.2. Results from In Vivo Studies

Chronic lymphocytic leukemia is characterized by the accumulation of malignant CD5^+^CD19^+^ B cells in peripheral blood, bone marrow, and secondary lymphoid organs. While a remarkably heterogeneous disease, CLL remains incurable with conventional therapy, and while many patients have a benign clinical course, others die within a short time from diagnosis. The proliferation of CLL cells in vivo occurs within lymphoid tissues in “proliferation centres”, where non-malignant cells of the microenvironment, mostly T-cells and mesenchymal stromal cells (MSCs) [24], provide numerous factors to support CLL survival and proliferation [25,26]. In addition, CLL cells can directly modulate T-cell function by the expression of cell-surface molecules and/or the production of soluble factors, including CD200 [27,28]. This type I transmembrane immunomodulatory molecule is known to be overexpressed in various malignancies [29,30,31], is shed from CLL cells in vitro, and is present in serum of CLL patients relative to healthy controls (sCD200) at levels which are related to the disease stage [23]. 

**Figure 1:**
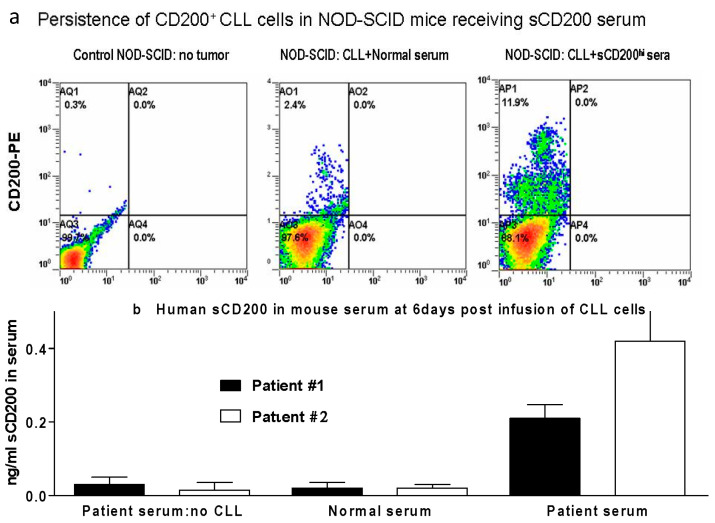
(**a**) 4/group NOD-SCID mice received 20 × 10^6^ purified CLL cells, iv, from a single patient. One group received 500 μL normal human serum iv (pooled from 10 healthy donors) 3 h later. An experimental group received 500 μL serum from the CLL patient donor (sCD200 level 4 ng/mL). Mice were sacrificed at d6 and splenocytes stained in FACS with anti-human CD200 mAb. (**b**) 3/group NOD-SCID received 20 × 10^6^ purified CLL cells iv and normal/autologous (patient) serum (the sCD200 levels in patient #1 and /#2 were 3.7 and 3.2 ng/mL, respectively). Other controls received patient serum only (no CLL). Day 6 sCD200 levels are shown.

In accordance with the significance of sCD200 present in CLL patient serum, we used a NOD-SCIDγ_c_^null^ mouse model to indicate that the exogenous delivery of such sCD200 plasma from CLL patients enhanced the seeding and growth of CLL from patient blood or spleen in vivo compared with normal human serum controls, with seeding to the peritoneal cavity, spleen, and blood. As expected, we observed variability in NOD-SCIDγ_c_^null^ mouse reconstitution, reflecting not just initial donor cell content but also sCD200 levels in plasma [23] (see Figure 1a,b below). 

CLL cells persisted in some recipients for as long as 75 days post splenocyte infusion with ki67^+^ cells (>70%), which were CD19^+^, still seen, reflecting ongoing proliferation in the CLL cells in this locale. In follow-up studies designed to explore the importance of sCD200 in CLL plasma in contributing to the engraftment of CLL cells in vivo, animals receiving CLL splenocytes and sCD200^hi^ serum also received a Fab anti-CD200 mAb (1B9), while other independent groups of mice received CLL splenocytes and CLL plasma that had been depleted of CD200 by passage through an anti-CD200 CNBr column. Both anti-CD200 mAb and the depletion of sCD200 from plasma attenuated the engraftment of CLL cells in vivo. Interestingly, the natural receptor for CD200, CD200R, was found to be mostly expressed on engrafted CD4^+^T-cells (but not CLL cells) in the spleen of engrafted mice, and the depletion of T-cells with OKT3 mAb in vivo also abrogated CLL engraftment despite the ongoing continuous infusion of sCD200^hi^ CLL serum [23]. These data suggest that CD200 enhanced CLL engraftment through an indirect action, targeting CD4^+^ cells in the microenvironment. More recently a vaccination approach to CLL (using PMA and ionomycin-stimulated CLLs as vaccine) [15] in concert with a blockade using anti-human CD200 antibody was shown to attenuate the local and metastatic spread of CLL cells in a NOD.SCID mouse model (below).

### 2.3. Results from In Vitro Studies

In a further extension of these in vivo studies, we used an in vitro model to examine the manner in which the TME might contribute to the regulation of CLL growth in vivo [19]. As noted above, it is thought that bone marrow mesenchymal stem cells (BMMSCs), derived from bone marrow and having the potential to differentiate into numerous other more committed cells, including osteoblasts, chondrocytes, and adipocytes, release multiple cytokines and chemokines potentially important to the regulation of tumor cell growth [25,26,32]. Amongst the cytokines identified in the literature as being of importance are IL-6, IL-8, and IL-17 [33,34]. IL-17A is a cytokine that induces the production of IL-6 and IL-8 in a variety of cells [35,36]. Using an in vitro model, we explored the role of IL-6, IL-8, and IL-17 in the survival of CLL cells, pertaining to their roles alone or in combination, in helping to explain the effect of BMMSCs on CLL growth both in vitro and in the NOD-SCIDγc^null^ (NSG) mouse model, and also compared the relationship between IL-17/IL-6 levels in CLL patient serum and clinical status [19]. We found that while BMMSCs themselves, or their culture supernatants, supported the growth/survival of CLL cells, hrIL-6 alone showed no effect on the survival of CLL cells in vitro. However, the addition of sIL-6R, at concentrations equivalent to those in BMMSCs supernatant, to the same hrIL-6 led to augmented CLL survival, with more pSTAT3 generated in CLL cells following this combined treatment, implying a role for both direct and transactivation by IL-6. Importantly, while we observed that IL-8 was also produced at high levels by BMMSCs, consistent with earlier reports [37], blocking IL-8 levels did not affect CLL survival [19]. The issue of levels of IL-8 reflecting prognosis in CLL and other cancers remains controversial [37,38]. 

The treatment of CLL cells, BMMSCs, or co-cultures of the two with human recombinant IL-17A, induced higher levels of IL-6 mRNA and protein in both CLL cells and BMMSCs. We concluded, given the effect of IL-6 on CLL survival discussed above, that IL-17 may be another important factor in CLL growth/survival. Indeed, mesenchymal stem cells promote tumor growth in allogeneic animals [39], and we had already established that BMMSCs (and hrIL-6) improved CLL engraftment in the NOD-SCIDγ_c_^null^ animal models, with mice receiving BMMSCs having more CLL cells engrafted in the spleen, bone marrow, and peritoneal cavity compared to mice receiving CLL cells alone [19]. Once again, this increase was attenuated by the IL-6R antagonist, Actemra. We found elevated levels of both IL-6 and IL-17 in CLL patients compared to the healthy controls. These levels were linearly correlated [19], possibly indicative of a positive feedback loop between the two cytokines with IL-17A directly influencing IL-6 production in CLL patients, and IL-6, an important cytokine in the differentiation of Th17 cells, in turn regulating levels of Th17, the major source of IL-17A [35]. 

### 2.4. A Model System Exploring Modified CLL Cells as a Tumor Vaccine

**Figure 2:**
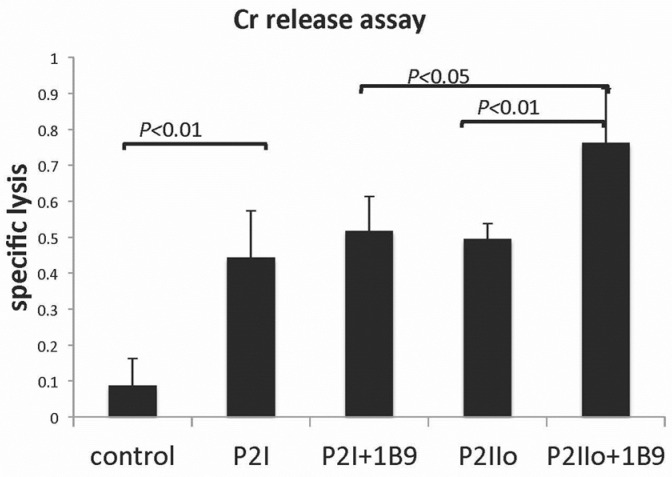
Tumor vaccine augmented CLL killing in vitro. All CLL cells used as tumor vaccine were effective in inducing specific autologous lysis in Cr release assay (*p* < 0.01). Blocking CD200 expression with anti-CD200 mAb, 1B9, on cells treated with P2I failed to increase killing. However, blocking CD200 expression with 1B9 on cells treated with P2Iio increased their ability to induce killing (*p* < 0.01). These same cells also induced higher autologous CLL cell lysis than cells treated with P2I alone (*p* < 0.05).

CLL cells, though malignant cells, retain at least some functions of normal B-cells. It is known that when normal B-cells are appropriately stimulated, they can present antigens, including any potential tumor antigens. This in turn suggests that stimulated CLL cells might be an excellent choice as a candidate tumor vaccine. We showed in a study designed to assess this possibility that following stimulation of CLL cells with Phorbol myristic acetate, IL-2, the TLR7 agonist imiquimod (P2I) and ionomycin (P2Iio), a marked increased expression of CD54 and CD83 occurred, indicative of B-cell activation and a transition to antigen-presenting cells [15]. However, simultaneous augmented expression of the known immunoregulatory molecule, CD200, was also seen after such stimulation. We next explored the effect of the stimulation of CLL cells with P2Iio, followed by the coating of cells with a non-depleting anti-CD200mAb, on the ability of those cells both to immunize PBL in vitro to become cytotoxic to CLL cells, and to protect NOD-SCIDγc^null^ mice from a subsequent CLL tumor challenge. We showed that this protocol was effective both in inducing CD8^+^ CTL able to lyse CLL cells in vitro (Figure 2), and in decreasing the tumor burden in vivo in the spleen and marrow of mice injected with CLL cells [15]. In a follow-up study, we pre-treated mice with a CD8-depleting antibody before vaccination with P2Iio/anti-CD200-coated cells, and showed that protection from CLL growth was abolished, implying a role for the blockade of CD200 expression on CLL cells as a component of a tumor vaccination strategy, which involved the induction of protection afforded by CD8 cytotoxic cells [15].

### 2.5. Supportive Data for CLL Studies in Animal Models in the Literature

As mentioned above, an anti-CD200 antibody led to the disruption of T-cell suppression as measured in autologous MLCs using CD40 ligand (CD40L)-stimulated CLL cells as antigen-presenting cells (APCs) [40], a mechanism inferred by Wong et al. in studies on NOD.SCID mice with CLL cells [23]. A similar mechanism has also been suggested as contributing to the mechanism behind increases in Foxp3^+^T-cell numbers in PTLD patients following the overexpression of CD200 [41]. 

Alternate mechanism(s) of action of the CD200:CD200 blockade in an AML model suggests a role for the augmented cytotoxicity of cytokine-induced killer cells against human myeloid leukemia blasts [42]—see also Figure 2 above. The CD200 blockade improved the regulation of AML growth by Tr1cells engineered to overexpress IL-10 [42], a phenomenon reminiscent of a claim that Ibrutinib, an inhibitor of Bruton’s tyrosine kinase (BTK) and IL-2-inducible T cell kinase (ITK), which is used in the treatment of CLL, alters CD200 expression and thus CLL-induced immunosuppression [43]. Finally, again using data derived from animal model systems, it has been suggested that CD200:CD200R interactions can control tumor growth through the regulation of the tumor microenvironment (TME) [44], an effect we have explored in more detail in a different (breast cancer) [20] model system discussed below.

Other groups have used mouse and zebrafish model systems to explore other human leukemias [45,46,47,48,49,50]. Studies of adult T-cell leukemia (ATL) caused by human T-cell leukemia virus type 1 (HTLV-1) infection have made use of transgenic and immunodeficient mice (HTLV-1 *Tax* and *HBZ* transgenic) primarily because of the low infectivity of HTLV-1 in mice [45]. These mice spontaneously develop tumors, allowing preclinical studies of candidate molecules for the treatment of ATL. In addition, HTLV-1-infected humanized mice with an established human immune system have been used to characterize cells in the early stages of HTLV-1 infection. In a similar vein, mouse xenotransplant models and transgenic animals have been of value in exploring drug development for human acute lymphoblastic leukemia (ALL) [46], including even exploring the effect of chemotherapy on cognition in ALL mouse models [47]. Zebrafish models of blood disorders, including myeloid and lymphoid malignancies, bone marrow failure syndromes, and immunodeficiencies have also advanced our understanding of these disorders, and allowed for translational research [48]. While we have focused on a NOD.SCID mouse model for human CLL research (see above), it is pertinent to acknowledge a body of work performed using other animal models [49], including transgenic mouse models (TCL1 mice) [50], for similar studies.

## 3. Use of Animal Models and In Vitro Studies in Breast Cancer (See Refs. [14,20] for Details)

### 3.1. Methodology… Breast Cancer Studies

*Mice:* Wild type (WT) BALB/c mice from Jax Labs, and CD200KO or CD200R1 knockout mice on a BALB/c background, are described elsewhere [14,51]. All mice were housed 5/cage in an accredited facility at UHN. Female mice were used at 8 weeks of age.

*Ethics Review:* All experimental studies described herein were approved by a local institutional review board, certified by the Canadian Council on Animal Care (protocol AUP#1.15).

*Monoclonal antibodies:* These, including rat Mab (1B9) to mouse CD200, are as described [52]. 

A rabbit Fab anti-CD200R1 antibody was prepared using a commercial kit (Pierce Protein Products, Rockford, IL, USA) following the immunization of rabbits with 500 μg mouse CD200R1 emulsified in Freund’s adjuvant. In independent studies, this antibody (1:1000 dilution) inhibited the binding (FACS analysis) of FITC-labeled mouse CD200 to Hek cells transduced to over-express murine CD200R1 [14]. In some in vitro studies (below), Fab rabbit anti-IL-6 or anti-IL-17 antibodies (Cedarlane Labs, ON, Canada) were used to explore cytokine roles in the TME in invasive tumor growth.

Human breast cancer is known to be a heterogeneous disease, with evidence that in some cases there is often evidence for protection from growth by a host immune response, while in other cases there is evidence that host inflammatory responses worsen disease outcome. Interestingly, two transplantable mouse (BALB/c) breast cancers, EMT6 and 4THM, seem to model these scenarios, with the former representing an immunogenic tumor, where host immunity can afford protection, while, in the latter, an inflammatory host reaction augments tumor growth. In the context of the notion that the activation of the CD200:CD200R axis was implicated in the attenuation of immune responses, we hypothesized that the growth of EMT6 (a CD200-expressing tumor) and 4THM (a CD200-tumor) would differ where transplantation occurred in mice with various manipulations of CD200/CD200R in mice or tumor cells.

Both EMT6 and 4THM tumors were grown following the injection of 2 × 10^5^ cells into the mammary fat pad of BALB/c female mice (WT or CD200^tg^, or CD200/CD200R KO). Lymph nodes draining the tumor (DLN) were harvested at the times indicated from the axillary nodes of tumor-bearing mice, with single-cell suspensions prepared after passing through a wire mesh. In vivo micro-metastases to draining lymph nodes (DLNs) were measured at 14 d using cells harvested from individual mice (5/group) and cloned at limiting dilution in microtitre plates for 21 days. Macro-metastases to the lung/liver were enumerated from mice in which primary tumors were resected at 14 d, with animals sacrificed 10 d later and organs visually inspected after fixation in Bouin’s solution. In other studies, tumors were resected from mice 14 d after injection, with mice then immunized ip with homologous irradiated (2000Rads) tumors cells (5 × 10^6^) mixed with MPLA (2 μg/mouse) in incomplete Freund’s adjuvant (IFA). These animals (5/group) were used as a source of tumor-immune DLN 14 d after immunization, or in studies designed to explore the efficacy of such vaccination strategies to protect from any subsequent challenge with further live tumor cells [14].

#### 3.1.1. 3D Culture System to Assess Tumor Invasion In Vitro 

A 3D culture system designed by Lonza (RAFT™ 3D Cell Culture System) was used in accordance with the supplier’s reagents, as described in detail elsewhere [20]. BMMSCs for inclusion in the collagen matrix were obtained from cultures of bone marrow cells, pooled from 3 control, non-tumor injected, donors of the indicated source and incubated for 12 d in 1 mM dexamethasone before trypsinization and washing. Gels were cast in 24-well plates and placed in an incubator. Each well contained 4 × 10^5^ stromal cells. Biocompatible hydrophilic RAFT absorbers supplied by the manufacturer were placed on the gels to remove the interstitial fluid from the collagen gels, the absorbers removed, and fresh culture medium added to each well before the plate was returned to the incubator. In some cases, the stromal cell suspension used also contained 10 × 10^6^/^mL^ DLN from naïve or tumor-immune mice. After 1 d of culture, the medium overlying the gel was removed and replaced with medium containing tumor cells (1 × 10^2^ of either EMT6 or 4THM origin). Six replicate wells were used/group

At 2.5, 5, and 7 days post initiation of culture, the upper medium layer was removed for 2 wells/group, rinsed, and tumor cells adherent to the surface collagen were released by trypsin. Viable tumor cell numbers were enumerated in the combined medium. To determine the frequency of tumor cells in the gel matrix phase the collagen gel was digested in each well with collagenase, the wells were thoroughly washed, an aliquot retained for cytokine ELISA and the remainder was used in a limiting dilution assay in microtitre plates with all wells containing 1 × 10^5^ irradiated BALB/c splenocyte feeder cells. The latter were added to improve the cloning efficiency at low numbers of tumor cells/well without having any effect on tumor cell viability/growth at non-limiting tumor cell numbers. These data allowed for the assessment of the kinetics of the growth of tumor cells detected by limiting dilution in the different phases using the various stromal cell feeder layers described in the gel matrix, for both EMT6 and 4THM tumors.

#### 3.1.2. ELISA Assay for TNFa, IL-6, IL-8, and IL-17 In Vivo and in 3D Cultures [20]

The aliquots of serum (2 μL/6 μL), or of the collagenase-treated gel phase or medium phase of the 3D cultures described above, were assayed in duplicate for TNFα, IL-6, IL-8, and IL-17 using commercial kits (BioLegend, San Diego, CA, USA). 

*Statistics:* Within experiments, comparison was made between groups using ANOVA, with subsequent paired Student’s *t*-tests as indicated. 

### 3.2. Results from In Vivo Studies

Early studies of the growth of tumor cells in mice with attenuation of CD200:CD200R, through either the silencing of tumor CD200 expression, or blocking host cell CD200R expression, showed that these manipulations did indeed decrease both local EMT6 tumor growth, and even invasion/metastasis to distant tissues [52,53], in accordance with our previous hypotheses. This was not the case when local/distant growth of the inflammatory breast cancer model, using 4THM cells, was studied [51]—see also [54]. Further analysis of host CD4/CD8 T-cell immunity, cytokine production, and the localized expansion of activated macrophages or myeloid-derived suppressor cells (MDSCs) led to further evidence of, and clarification of, how differences in adaptive immunity and host inflammation could help explain these data. Additional expansion of such studies to include analysis of miRNA and protein expression data from serum-derived exosomes of different tumor-bearing mice suggested a key role for a number of previously described molecules, including TGFβ and various inflammatory cytokines, in the regulation of metastatic tumor growth in these models [55]. These studies highlighted novel approaches that might be of clinical utility, as investigated in preliminary reports [56,57].

### 3.3. A Model System Exploring Breast Cancer Cells as a Tumor Vaccine

**Figure 3:**
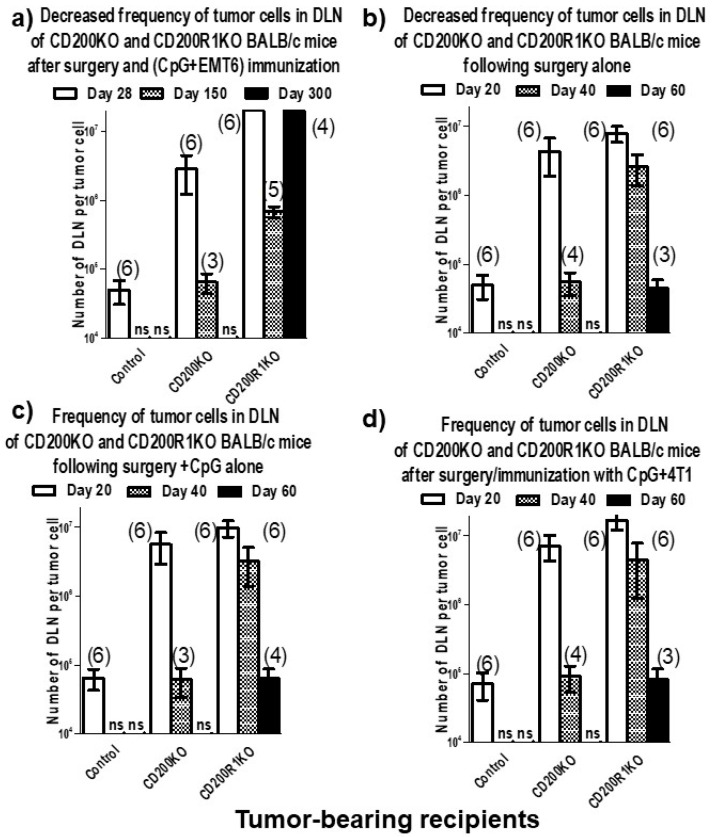
Decreased frequency of tumor cells cloned from DLN cells of CD200KO or CD200R1KO mice, compared with control BALB/c, is most pronounced in mice immunized with (CpG+EMT6), but not CpG alone or CpG+4T1 after surgical resection, (compare panels (**a**–**d**)). DLN cells were harvested from individual mice in each group at the time points shown and cultured under limiting dilution for 3 weeks to assess the frequency of tumor cells. Data show arithmetic means (+SD) for each group. The number of survivors at each time point is indicated besides each bar; ns = no survivors.

In preliminary studies, we found that CD200R1KO mice with EMT6 tumors could be cured of tumor growth following surgical resection and immunization with irradiated EMT6 cells and CpG, while wild-type (WT) animals developed pulmonary and liver metastases within 30 days of surgery [14]—see Figure 3. WT mice could be cured of EMT6 growth by surgical resection in the same fashion as CD200RKO mice if primary tumor resection was followed by iv infusions of Fab anti-CD200R1 along with CpG/tumor cell immunization [14]. Once again, growth of the highly metastatic 4THM tumor occurred in both CD200RKO mice and WT mice after primary tumor resection along with CpG/tumor cell immunization, even when, in WT mice, resection was followed by iv infusions of Fab anti-CD200R1. Metastasis was both macroscopically (lung/liver nodules) and microscopically followed in all cases by cloning tumor cells at limiting dilution in vitro from draining lymph nodes (DLN) harvested at surgery. To explore the role of the development of an effective host immune response in the different outcomes in these scenarios, we compared local/metastatic tumor growth in mice receiving the treatments described with that seen in similar mice but receiving not tumor cell vaccination but four courses of combination treatment with anti-VEGF and paclitaxel [14]. 

Interestingly, in WT mice receiving EMT6 and Fab anti-CD200R, no tumor cells were detectable after immunotherapy (tumor cell vaccination), and the CD4+ cells produced increased TNFα/IL-2/IFNγ on stimulation with EMT6 in vitro, as had been seen already in CD200RKO mice. As previously reported, no long-term cure was seen following surgery/immunotherapy of 4THM, with both microscopic (tumors in DLN at limiting dilution) and macroscopic metastases present within 14 d of surgery. However, we did observe that chemotherapy with Rabbit anti-VEGF (AbCam, Boston, MA, USA (ab46154)) and paclitaxel (Taxol 10 mg/Kg) could lead to attenuated growth/metastases in 4THM tumor-bearers and produce a decline in lung/liver metastases. This same chemotherapeutic treatment in EMT6 tumor bearers resulted in no detectable DLN metastases in EMT6 tumor-bearing mice, as had been seen after immunotherapy. However, following chemotherapy, the mice showed no significantly increased cytokine production after restimulation with EMT6 in vitro, in contrast to cell used from immunotherapy-treated mice. In addition, EMT6 mice receiving immunotherapy were resistant to a subsequent re-challenge with EMT6 tumor cells, but not those receiving curative chemotherapy. Furthermore, anti-CD4 treatment caused tumor recurrence after immunotherapy in EMT6-injected mice, but produced no apparent effect in either EMT6 or 4THM tumor bearers after chemotherapy treatment [14]. We concluded from these exhaustive studies that while chemotherapy might be effective in “curing” the growth/metastasis of both the poorly immunogenic 4THM tumor, and an immunogenic tumor in mice (EMT6), nevertheless, tumor vaccination immunotherapy was an ineffective treatment regime for the cure of 4THM. Furthermore, only immunotherapy, but not chemotherapy, enhanced CD4^+^ immunity and afforded long-term control of breast cancer growth and resistance to new tumor foci for EMT6 tumors.

### 3.4. Results from In Vitro Studies

As detailed above, a number of in vivo studies have suggested a role for CD200:CD200R interactions in the control of tumor invasion. Given the important role of the local tumor microenvironment on this issue, we used a 2-phase 3D culture system in order to attempt to mimic the in vivo model systems previously discussed [58]. In this model, bone-marrow-derived stromal cells (BMMSCs) from 12-day pre-cultures of T-depleted bone marrow cells were embedded in a collagen matrix, and tumor cells were allowed to infiltrate this matrix from a liquid culture layer above. Mesenchymal stem cells as well as tumor-associated stroma cells (TASCs) are known to be important components in the microenvironment of tumors and BM-MSCs have been shown to modulate cell growth and survival through cell–cell contact and cytokine production, including IL-6, IL-17, and tumor necrosis factor (TNF) [59]. Further regulation of tumor invasion was explored after inclusion, with BMMSCs in the collagen gel, of CD4^+^-enriched T-cells from DLN of tumor-immunized mice. The latter were derived from mice whose tumors were resected (14 d post inoculation into the fat pad), with the animals subsequently immunized with the corresponding irradiated cells admixed with CpG as adjuvant 2 d after surgery, and DLN harvested 14 d later. Tumor cells invading the collagen gels were measured by limiting dilution analysis after 7 d of culture on the collagen gel substratum, with tumor cells released by collagenase digestion. Cytokines were also measured in the gel after this treatment using commercial ELISAs.

**Figure 4:**
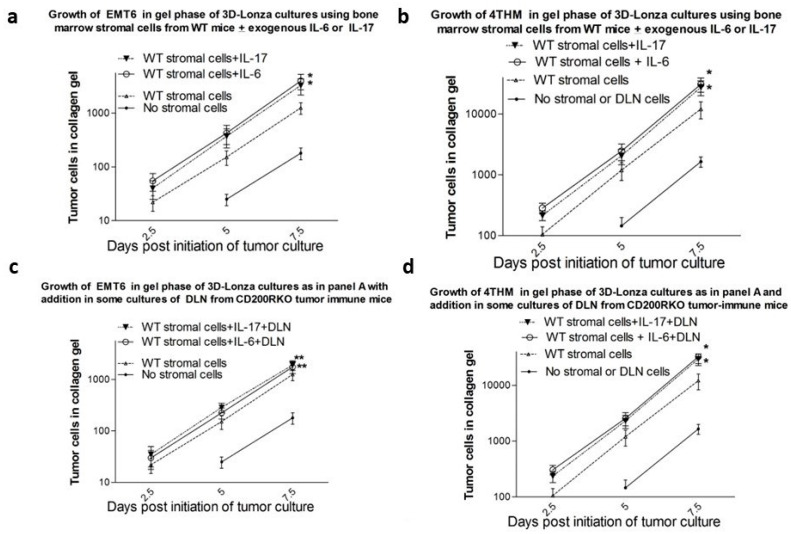
Tumor cells detectable in the collagen gel phase of cultures where 4 × 10^5^ bone marrow-derived stromal cells (BMSCs) from WT mice were included in the collagen gels with/without 150 pg/mL recombinant IL-6 or IL-17. All cultures were initiated by seeding 100 EMT6 or 4THM tumor cells into the liquid culture overlying the collagen gels with data for tumor growth pooled from three independent studies, using six cultures per group, with two harvested at each time point. Note in panels c/d, all groups also contained DLN cells from immunized CD200R1KO mice (see text). * *p* < 0.05 compared with BMSCs only (no cytokines) in gel (Mann-Whitney U-test). ** *p* < 0.05 compared with equivalent group without DLN. Reproduced with permission—see [20].

This novel in vitro system readily discriminated between invasion of TMT6 and 4THM breast tumors, a phenomenon we had previously reported in vivo—see above. Amongst the factors controlling this differential invasiveness was a different response to CD200:CD200R interactions. ***Importantly, no such discrimination was seen by comparing growth in liquid cultures, and no significant effect of CD200:CD200R interactions occurred in liquid cultures.*** Data from this in vitro system were found to parallel many of the in vivo results previously reported. High levels of IL-6 and IL-17 were observed in collagen gels containing stromal cells and 4THM immune DLN cells, in association with augmented tumor invasion of 4THM, an effect abolished by the inclusion of Fab rabbit anti-IL-6 or anti-IL-17 antibodies into the collagen matrix [20]. In contrast, while EMT6-immune mice showed elevated serum levels of IL-6, and augmented tumor invasion after the inclusion of recombinant IL-6 or IL-17 in the collagen gel matrix, the addition of EMT6-immune DLN cells attenuated tumor invasion despite the inclusion of exogenous IL-6/IL-17 in the gel matrix (Figure 4), an effect abolished by the T depletion of DLN. 

Consistent with current thinking on the importance of the tumor microenvironment to tumor growth and tumor invasion [60,61,62,63,64], we found that infiltration of tumor cells into a 3D collagen matrix over 7 d in culture was promoted by the presence of stromal cells in the culture matrix. While tumor metastasis in vivo differed for 4THM and EMT6 tumors in WT, CD200KO, and CD200R1KO mice (see above), all stromal cells were equally efficacious in promoting the tumor invasion of tumor cells into the matrix, implying a role for stromal cells in tumor invasion, which is independent of the CD200:CD200R axis. One such mechanism that was implicated in the disparity of metastasis in vivo was highlighted by studies using DLN from various tumor-immune mice along with stromal cells in the collagen gel matrix. This led to a marked enhancement of tumor invasion of 4THM cells, with attenuation of tumor invasion of EMT6 in the presence of DLN of CD200KO and CD200R1KO mice, both phenomena which were reflected in previous in vivo studies. Augmented IL-6 and IL-17, seen in 4THM tumor sera and in collagen gels, were thought to be a likely explanation for the increased 4THM tumor invasion, and indeed, the increase was abolished by anti-IL-6/IL-17 antibodies. However, IL-6 and IL-17 expression was also increased in DLN containing collagen matrices with EMT6 tumor cells, yet in this case we saw decreased tumor invasion, despite prior in vivo evidence that IL-6/Il-17 played a role in the enhanced metastasis of EMT6 cells in vivo [55]. We hypothesized that while IL-6/IL-17 were primary mediators of tumor invasion for both 4THM and EMT6 tumor cells, an independent function of the added DLN cells could lead to the attenuation of EMT6 tumor invasion, and the data implicated a role for non T depleted (but not T depleted) DLN cells from CD200RKO mice in this function. 

We suggested that future use of this novel in vitro model might help identify the cellular source of IL-6 and IL-17 in mixed cultures of stromal + DLN cells, as well as the mechanisms implicated in the augmented production of those cytokines in the presence of tumor cells. Suitable approaches might involve drug-targeting studies, and/or using cells (stroma/DLN) from transgenic/knockout mice to assess the contribution of these cell sources. The mechanism(s) implicated in the antagonism of IL-6/IL-17-facilitated tumor invasion of EMT6 by T-cells in DLN cells from CD200R1KO mice noted above may reflect a direct action on the tumor cells themselves, or an indirect action on a cytokine or chemokine-induced pathway, although there was no evidence from our studies that DLN cells simply acted in a cytostatic or cytostasis role. It remains unclear why and how DLN cells do not antagonize cytokine-induced tumor invasion for the 4THM tumor. It is known that miRNA expression profiles differ in cells derived from WT, CD200KO and CD200R1KO mice bearing EMT6/4THM tumors [55], and a difference in miRNA-mediated regulation and the relative importance of inflammatory cytokines and the epithelial-to-mesenchymal transition to the growth/tumor metastasis of 4THM and EMT6, may indeed be responsible for some of the differences seen [65,66]. Cancer-associated fibroblasts are themselves known to be a rich source of exosomal miRNAs [67,68], and can alter the expression of a number of molecules, including IL-6 and those of the Wnt pathway [69,70], which can contribute to tumor invasion. miRNAs have been reported to alter the expression of molecules, e.g., PD-L1 [71], which, like CD200:CD200R, are known members of the checkpoint inhibitor family, which can also modulate host anti-tumor responses [12]. A further application of this in vitro model might be to use tissue-specific stromal elements in the culture to explore whether this might help explain the observed organ-specific metastasis seen for tumors growing in vivo [72].

### 3.5. Supportive and Cautionary Data for Breast Cancer Studies in Animal Models from the Literature, including Other Solid Tumor Models

It is acknowledged that the expression of CD200 can play a pro-tumorigenic role in various malignant tumors, both hematopoietic and solid tumors [73], often through indirect effects mediated by an altered tumor microenvironment (TME) [44,54]. There is now a growing body of evidence, driven both by clinical research and animal experimentation, that this potentially represents an untapped therapy in solid cancer biology [12], with evidence for the overexpression of *CD200* in bone, lung, and liver metastatic tissues from patients with aggressive breast cancer compared with adjacent noncancerous breast tissues from those with non-metastatic breast cancer [74]; in non-small-cell lung cancer patients and in one-third of those with lung large-cell neuroendocrine carcinoma (LCNEC) [75]; in peritumoral stroma from patients with HCC [76]; in poorly differentiated laryngeal cancer, human pancreatic ductal adenocarcinoma (PDAC), and human clear cell renal cell carcinoma (ccRCC) [77,78,79]; in various types of skin cancer, including cutaneous squamous cell carcinoma (cSCC) [80] and Merkel cell carcinoma (MCC) [81]; and in various subgroups of human brain tumors, including glioblastoma, medulloblastoma, ependymoma, and neuroblastoma [82,83].

In the SCC model, Khan et al. [80] had already reported a pro-metastatic role for the CD200-CD200R axis, which was independent of direct T-cell suppression and likely involved modulation of the function of infiltrating myeloid cells. In a newer study, they identified the upregulation of cysteine protease cathepsin K (Ctsk) in CD200^+^ cSCCs. Using a CD200R^+^ myeloid cell-cSCC co-culture system they showed that induction of Ctsk was dependent on CD200-CD200R interactions, implying that Ctsk might be an important target gene in the cSCC tumor microenvironment. Indeed the inhibition of Ctsk, but not matrix metalloproteinases (MMP), was found to block cSCC cell migration in vitro. In support of these studies, targeted disruption of CD200 expression in cSCC tumor cells and Ctsk pharmacological inhibition reduced cSCC metastasis in vivo. In the Merkel cell tumor xenograft model, high *CD200* mRNA expression was found in MCC tumors, and CD200 immunostaining was demonstrated on >95% of MCC tumors, with CD200R^+^ myeloid cells present in the MCC tumor microenvironment. MCC-associated macrophages had a two-fold higher CD163:CD68 staining ratio than the controls, suggestive of an immunosuppressive M2 phenotype, and also consistent with the increased densities of FOXP3+ regulatory T-cells seen. The infusion of blocking anti-CD200 antibody to MCC xenograft mice led to increased targeting to the tumor, which the authors suggested might portend a novel immunotherapy for MCC, independent of the PD-1/PD-L1 blockade.

Many (most) of the effects of CD200 are mediated following binding to a CD200 receptor, known to be expressed on cells of the myeloid lineage, as well as B-cells and activated T-cell subsets. CD200R interacts with the NH2-terminal domain CD200 through its NH2-terminal domain, with the subsequent phosphorylation of a tyrosine motif in the CD200R cytoplasmic tail; binding and phosphorylation of the adaptor proteins tyrosine kinase 1 (DOK-1) and tyrosine kinase 2 (DOK-2) with resultant binding of SH2-containing inositol phosphatase (SHIP) to DOK-2; and finally, recruitment of Ras GTPase-activating protein (RasGAP) and inhibition of the MAPK signaling pathway [84,85]. These processes ultimately lead to altered pro-/anti-inflammatory cytokine release and immune cell activation [85,86]. It has been argued that it is this interaction between CD200 on SCC keratinocytes and CD200R on myeloid-derived suppressor cells that in turn promotes the metastasis of SCC [87]. More recently, the notion that altered tumor growth might depend on the relative affinities of the interaction (in the TME) between CD200 and CD200R expressed on M2-type macrophages compared with those on M1-type macrophages has been raised [88]. It is studies such as these that have fostered exploration of the manipulation of the CD200:CD200R interaction as an adjunctive checkpoint therapy in clinical cancer [12]. 

In a recent study using neuroblastoma and melanoma mouse models to evaluate the roles of CD200R signaling in tumor growth and immunity, it was reported (in accord with the EMT6 breast cancer model discussed above) that CD200RKO mice were significantly more potent in rejecting these CD200^+^ tumors [85]. Tumors from CD200RKO mice had more infiltrating CD4^+^ and CD8^+^ T-cells, and NK cells and decreased neutrophils – see also [53]. Antibody depletion showed that immune effector cells were important in inhibiting tumor growth. The mechanism behind these effects was hypothesized to involve CD200:CD200R-mediated regulation of the expression of chemokines in tumor-associated myeloid cells, which, in the absence of CD200R engagement, produced CCL24 with the increased infiltration of eosinophils, contributing to anti-tumor activity. 

By far the most extensive studies of CD200:CD200R-mediated immunotherapy in animal models of CNS tumors have come from Olin’s laboratory [89,90,91]. Many of these studies have used a combination approach, which included inhibition of immune checkpoint proteins in the tumor microenvironment and tumor lysate-based vaccination strategies [89]. In a dog model of high-grade glioma, a synthetic peptide that targeted the immune checkpoint protein, CD200, enhanced the functional ability of antigen-presenting cells to activate T-cells for an anti-glioma response [90]. Using this CD200-directed peptide before the subcutaneous delivery of an autologous tumor lysate vaccine doubled survival relative to animals given tumor lysate alone, and was associated with the suppressed expression of inhibitory CD200R receptor on T-cells, and APCs, thereby overriding the CD200-mediated suppression of a glioblastoma microenvironment overexpressing immunosuppressive CD200 protein. 

Further studies [91] have characterized the possible mechanism(s) by which this CD200 peptide ligand (CD200AR-L) functions following presumed binding to an (as yet) uncharacterized CD200 immune-checkpoint activation receptor (CD200AR)—see also [92]. Studies have investigated in particular the activation of the DAP10&12 pathways using both in vitro studies of transcription, protein, and phosphorylation expression, and in vivo loss of function studies with inhibitors to select signaling molecules. CD200AR-L/CD200AR binding was reported to induce a rapid activation of the DAP10&12 pathways, followed by a decrease in activity within 30 min, and then with a reactivation via a positive feedback loop. In vivo studies using DAP10&12KO mice showed that DAP10, but not DAP12, expression was crucial for tumor growth control. As already reported [90], it was the combination of CD200AR-L with an immune-stimulatory gene therapy (tumor lysate vaccine), which increased median survival, and long-term survivors. It is worth noting a caveat to both these and the EMT6 studies discussed earlier. Again, using animal models (EMT6, a Lewis Lung Carcinoma model, LCC, and melanoma), Pilch et al. reported that while agonistic CD200R antibodies (two were tested) reached tumors, they had **no** significant impact on tumor growth and only a minor effect on the infiltration of immune myeloid cells [93]. These effects were reproduced using two different anti-CD200R clones. In contrast, and as reported by many others, CD200-deficiency decreased melanoma tumor burden [93], while both endogenous or tumor-expressed CD200 restored the growth of metastatic melanoma foci. These authors, at least, concluded that the blockade of endogenous CD200 can prevent the tumorigenic effect of CD200R-expressing myeloid cells in the tumor microenvironment, but ***agonistic anti-CD200R may not necessarily produce such an effect.***

An interesting complexity to the CD200:CD200R story comes from recent findings that human carcinoma tissues express not only the full-length CD200 but also a truncated form, CD200tr, previously reported in other models as a likely antagonist to CD200 [94,95]. Using parent rat C6 glioma cell lines, and cells transduced to express either CD200 or CD200tr, a comparison of growth in neonatal Wistar rat forebrain parenchyma was monitored (all lines equivalently grew in vitro). Rats transplanted with C6-CD200tr cells survived for longer periods than those receiving C6 or C6-CD200 cells, and C6-CD200tr tumors were smaller with multiple apoptotic cells in the tumor mass. Tumor-associated macrophages (TAMs) in C6-CD200tr tumors had dendritic cell (DC)-like morphology and CD86 expression, and there were more CD3^+^, CD4^+^, and CD8^+^ cells, and increased expression of granzyme and perforin in C6-CD200tr tumors. When TAMs from original C6 tumors were co-cultured with C6-CD200tr cells, an increased expression of DC markers occurred. The authors suggested that the expression of CD200tr led to the activation of TAMs to become DC-like antigen-presenting cells, with activation of CD8^+^ CTL and apoptotic elimination of tumor cells. More recent findings in another human xenograft model of head and neck cancers [96] showed that in mice transplanted with head and neck squamous cell carcinoma (HNSCC), cells overexpressing CD200 had attenuated tumor growth when treated with adenovirus-expressing soluble CD200R-Ig. This seemed to correlate with the elimination of the induction of M2-like cells, increased recruitment of regulatory T-cells, and decreased numbers of CD8^+^ T-cells associated with CD200 SCCs. 

A final proviso should be added to these studies given the recent report from Talebian et al., who were driven to investigate the impact of the CD200 blockade in what is thought to be a more clinically relevant model for human melanoma, namely CD200^+^ Yumm1.7cells [97]. Yumm1.7 cells are known to express Braf/Pten mutations such as human melanoma. Yumm1.7 tumors were found to grow faster in CD200RKO mice compared to wild-type mice, and detailed analysis showed that tumors from both CD200RKO or anti-CD200-treated mice showed decreased immune cell components and reduced TCR clonality compared to tumors from untreated wild-type mice. The T-cell effector function was also impaired, with reduced numbers of IFN-γ^+^ and TNF-α^+^ T-cells and an upregulation of the expression of the CCL8 (macrophage-derived) gene. Co-culture studies using Yumm1.7 tumor cells and bone-marrow-derived macrophages (BMDM) from WT or CD200RKO mice confirmed the increased expression of macrophage CCL8 in the absence of CD200-CD200R interactions. *Finally, this group reported that anti-CD200 therapy was ineffective alone, or in combination with checkpoint inhibitors, including anti-PD-1 and anti-CTLA4, in inhibiting Yumm1.7 tumor growth*. They concluded that these studies should prompt caution in using the blockade of CD200 as an immunotherapy for melanoma. 

## 4. Summary

The above discussion has provided an overview of the use of animal models that my laboratory has adapted to study immunoregulation in two discrete models of malignancy, namely CLL and breast cancer, and the role played by the CD200:CD200R axis in that regulation. Also highlighted were many of the ongoing unresolved controversies. It should be noted, if it is not already self-evident, that the development of cancer immunotherapy, and in particular the study of immune checkpoint inhibition in cancer therapy, has followed many of the approaches/methodologies used herein [10,11,98,99,100,101,102]. Where relevant, similar research by other groups, again using animal studies, have been cited, both in support of, and occasionally contradictory to, our own findings. No significant review of the growing body of clinical literature available, which supports many of these observations, has been attempted, but the interested reader is referred to the following references as an introduction to this rapidly growing field [16,103,104,105,106,107]. There can be little doubt that ongoing use of animal models will in the future, as in the past, drive expansion of this vast area of translational research.

## Data Availability

All data are contained within the article.
